# Evolution of conserved secondary structures and their function in transcriptional regulation networks

**DOI:** 10.1186/1471-2164-9-520

**Published:** 2008-11-02

**Authors:** Hai-Bing Xie, David M Irwin, Ya-Ping Zhang

**Affiliations:** 1 State Key Laboratory of Genetic Resource and Evolution, Kunming Institute of Zoology, Kunming 650223, PR China; 2 Laboratory for Conservation and Utilization of Bio-resources, Yunnan University, Kunming 650091, PR China; 3 The Graduate School, Chinese Academy of Sciences, Beijing 100049, PR China; 4 Department of Laboratory Medicine and Pathobiology, University of Toronto, 100 College Street, Toronto, Ont., M5G 1L5, Canada

## Abstract

**Background:**

Many conserved secondary structures have been identified within conserved elements in the human genome, but only a small fraction of them are known to be functional RNAs. The evolutionary variations of these conserved secondary structures in human populations and their biological functions have not been fully studied.

**Results:**

We searched for polymorphisms within conserved secondary structures and identified a number of SNPs within these elements even though they are highly conserved among species. The density of SNPs in conserved secondary structures is about 65% of that of their flanking, non-conserved, sequences. Classification of sites as stems or as loops/bulges revealed that the density of SNPs in stems is about 62% of that found in loops/bulges. Analysis of derived allele frequency data indicates that sites in stems are under stronger evolutionary constraint than sites in loops/bulges. Intergenic conserved secondary structures tend to associate with transcription factor-encoding genes with genetic distance being the measure of regulator-gene associations. A substantial fraction of intergenic conserved secondary structures overlap characterized binding sites for multiple transcription factors.

**Conclusion:**

Strong purifying selection implies that secondary structures are probably important carriers of biological functions for conserved sequences. The overlap between intergenic conserved secondary structures and transcription factor binding sites further suggests that intergenic conserved secondary structures have essential roles in directing gene expression in transcriptional regulation networks.

## Background

Conserved genomic elements are shared by a wide spectrum of organisms, and with the increased availability of sequenced genomes, it is now feasible to identify these elements by implementing comparative genomic analysis with highly divergent species. A series of studies have focused on the identification of conserved elements in the human genome, and have revealed that about 5% of the genome is composed of these conserved elements [1,2]. The precise number of conserved elements in a genome identified in different studies varies though, due to the specific search criteria used and the degree of divergence between the genomes analyzed [1,3,4]. The primary criteria for the identification were largely based on the sequence identity. For example, Bejerano *et al*. defined 481 ultraconserved elements as sequences at least 200 base pairs showing 100% identity in human-mouse and human-rat genomic comparisons [3]. An alternative strategy was used by Cooper *et al*. who calculated "rejected substitutions" (RS) value for sequences, where RS is computed by comparing the number of observed substitutions to that estimated if the sequences were evolving neutrally, thus sequences with high RS values show high identity, and with a threshold of 8.5 RS this method achieved about 95% confidence in the identification of conserved elements [1]. In the human genome, conserved elements range in size from dozens to thousands of base pairs in length [1]. While some elements overlap protein coding sequences, most are located in intergenic and intronic regions of the genome [5]. Even in simpler organisms, conserved elements are an important component of their genomes [6]. Searches in vertebrate, insect, worm and yeast genomes have found that as genome sizes increases, a larger fraction of the conserved elements are located outside of the exons of protein coding genes [6].

Despite the well documented existence of conserved elements, the significance of these sequences remains largely unknown [7]. Evidence suggests that conserved elements represent a variety of different types of DNA sequences [8]. Some families of ancient repetitive sequences have been found to be under strong purifying selection and are conserved among many species [9]. Some conserved elements have been identified as genes encoding microRNAs, for example the microRNAs in insects [10]. The highest number of microRNA genes estimated for metazoans and plants is about 2,500, with only about 1,000 of these genes being estimated in the humans [10], thus microRNA genes can only represent a tiny fraction of the conserved elements. Other attempts have been made to characterize the potential functions of conserved sequences, most of which document a statistically significant association between conserved elements and gene families for transcription factors and developmental regulators [3,4,11,12]. Experimental essays have been done to characterize the transcriptional activities of only a handful of conserved elements, with a few being found capable of driving the expressions of proximal genes [11,13,14], thus strongly suggesting that these conserved elements may have enhancer activity. Conserved elements may confer their regulatory activity over great genomic distances. A recent analysis, based on duplicated conserved elements, indicated that the distance of regulatory activity of conserved elements can vary dramatically, with more than half of the elements regulating target genes that are more than 250 kb, and as much as 2 Mb, away [15]. In addition, Frazer *et al*., in a study of conserved elements in the *SIM2 *interval, uncovered an additive effect of adjacent elements on promoting gene expression [13], suggesting that some of the conserved elements function together despite the great distances that separate them from their target genes. The distance between highly conserved elements is also conserved [16]. Less variation in distance between conserved elements is observed compared to the distances between protein coding sequences in human-mouse and human-dog genome pairs. This observation implies that the interval space size or orientation may be important for the co-function of these elements [16]. Abnormal action of conserved element can lead to genetic diseases [8]. Many developmental diseases have been characterized as being due to the malfunction of conserved noncoding sequences, including preaxial polydactyly [17], blepharophimosis syndrome [18], and Van Buchem disease [19].

Many conserved secondary structures (CSSs) were identified in the human genome by using an eight-way genome-wide alignment. Some of the CSSs identified in this alignment have been identified as known functional RNAs, such as microRNAs, histone 3'-UTR stem-loops, and some genetic recoding elements [20]. In insects, a conserved element with a secondary structure has been implicated in the control of alternative mRNA splicing [21], thus potentially some of the human elements may have similar roles. However, the functions of most of the identified CSSs remain unknown. In this study, we analyzed the evolutionary constraint acting upon CSSs using data from SNPs and demonstrated that about 1/3 of the mutations in CSSs were eliminated by selection in human populations and that sites in the stems of the predicted secondary structures are under stronger constraint than sites in loops/bulges. A substantial number of intergenic CSSs overlap the binding sites for transcription factors and are located proximal to transcription factor-encoding genes, thus we speculate that they may function in transcriptional regulation networks. We suggest that a substantial portion of intergenic CSSs function as cis-regulators and that the structural conservation is partially attributed to steric requirement for interacting with transcription factors.

## Results

We initiated this study by reanalyzing the CSSs data originally produced by Pedersen *et al*. [20]. CSSs were predicted with EvoFold program [20], from sequences defined as conserved sequences by the PhastCons method [6] from a whole genomic alignment generated by the MULTIZ program [22] using the human, chimpanzee, mouse, rat, dog, chicken, zebra fish, and puffer fish genomes. Only long secondary structures (at least 15 pairing bases) were included in our analysis, with a focus on examining polymorphisms within them and the associations between intergenic CSSs and their neighboring genes. Of the total of 9404 long CSSs, 4473 are located in intergenic regions, 2690 are located inside intronic regions, 1428 overlap within protein coding sequences (CDSs), and the remaining 783 are located in untranslated regions (UTRs) of genes. To measure the evolutionary constraint acting upon CSSs, SNPs were used to determine the polymorphism density and derived allele frequencies. Data on SNPs and recombination rates were obtained from the HapMap project and from the dbSNP database. Genetic distance between SNPs spanning intergenic CSSs and flanking genes were calculated using the recombination rate information and was used for investigating the associations between CSSs and their flanking genes.

### Evolutionary constraint in the CSSs

**Figure 1:**
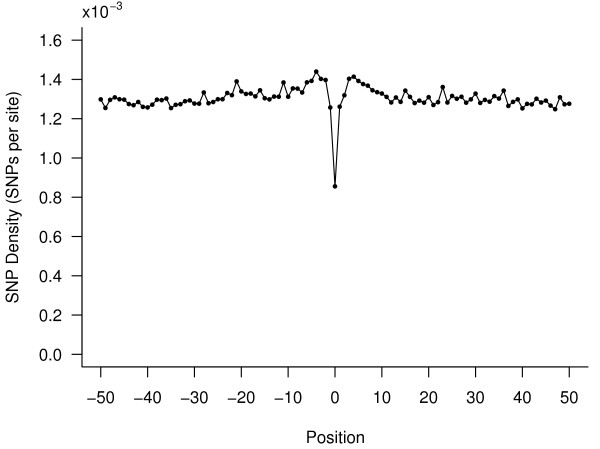
**SNP density is lower in CSSs than in flanking sequences**. SNP density was calculated in 200 bp moving windows, without overlap, centered on the CSSs with 50 windows chosen on each side of the CSSs. The SNP density at position 0 indicates the SNP density in the CSSs. SNP density in CSSs (0.86 SNP/kb) is about 2/3 of that of their flanking sequences (1.30 SNP/kb).

CSSs were identified by using a human-referenced eight-way genome-wide alignment, but the evolutionary variation of the elements in human populations is unknown. We used SNP density to measure the level of constraints on CSSs and their flanking sequences. To determine variation in SNP density between CSSs and their flanking sequences, SNPs were mapped to CSSs and their flanking sequences. A total of 746 SNPs were mapped to CSSs. CSSs had much lower SNP density compared to their flanking sequences, as shown in Fig. 1. The density of SNPs in CSSs (0.86 SNP/kb) is about 65% of that of their flanking sequences (1.30 SNP/kb). This decreased density is much lower than the 82% density which had previously been observed for conserved elements compared to nonconserved sequences [23]. Minimal variation in SNP densities was observed in the flanking sequences (Fig. 1). Since sequences on both sides of CSSs have higher SNP densities than CSS sequences, a difference in mutation rate does not appear to be a likely reason for the observed difference in SNP density. Since the flanking sequences appear to be evolving largely at a neutral rate, the most plausible explanation for the observed decrease in SNP density is that about 35% of the mutations that occur in CSSs are harmful and have been removed by selection. This explanation would imply that CSSs function in fundamental biological roles where a significant fraction of the mutations within them have deleterious effects and are removed by natural selection within human populations.

To investigate whether the SNP density varies within CSSs, we classified nucleotide sites of the CSSs as being in predicted stems or loops/bulges according to their positions in the structural folding predicted by EvoFold. A total of 559,960 nucleotides mapped to stems with 311,897 nucleotides mapped to loops/bulges of CSSs. Of the 746 SNPs located within CSSs, 392 SNPs mapped to stems and 354 SNPs to loops/bugles, demonstrating that stems have a much lower SNP density (0.70 SNP/kb), of about 62% of the density of loops/bulges (1.13 SNP/kb). This result implies that a very large fraction of the mutations that occur in stems appear to be deleterious and are removed by selection within the human populations, suggesting that mutations in the stems of secondary structures have a greater impact compared to mutations in loops/bulges in the function of CSSs. Indirectly, this data also further support the existence of CSSs in the human genome, as we would not expect to observe differences in SNP density if the secondary structures were simply due to false positive folding. SNP density of loops/bulges is still lower than that of the flanking sequences, suggesting that sites in the loops/bulges are also constrained.

**Figure 2:**
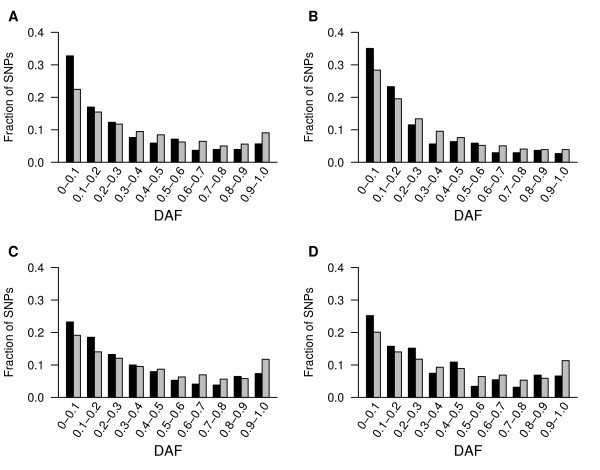
**DAFs are lower for SNPs in CSSs than in flanking sequences**. Derived allele frequencies (DAFs) were calculated in bin frequencies of width 0.1 for four HapMap populations: A: CEU; B: YRI; C: JPT; D: CHB. Black bars represent data for SNPs in CSSs and gray bars indicate their flanking sequences.

**Figure 3:**
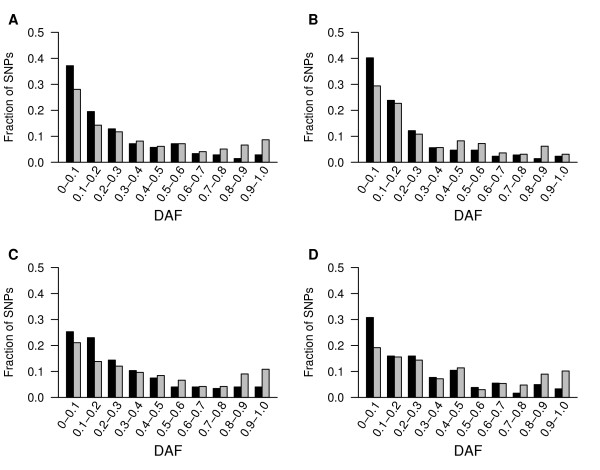
**DAFs are lower for SNPs in the stems than in the loops/bulges of CSSs**. Derived allele frequencies (DAFs) were calculated for predicted stems and loops/bulges and grouped in frequency bins of width 0.1 for four HapMap populations: A: CEU; B: YRI; C: JPT; D: CHB. Black and gray bars represent data for SNPs on stems and on loops/bulges of CSSs.

To provide further insights into the evolutionary constraint acting on CSSs, we examined the frequency distribution of the SNPs. Here, each SNP was classed as one of two alleles, namely ancestral and derived alleles. The ancestral allele is the allele inherited from the common ancestor of human and chimpanzee, while the derived allele is the allele that has been generated by mutation of the ancestral allele within human populations since the divergence from the common ancestor. Derived allele frequency (DAF) indicates the frequency of the derived allele. Selective constraint can be directly viewed by examining the frequency distribution of derived alleles of SNPs within populations, where differing mutation rates should not affect the frequency distribution [24]. We first compared the DAFs of SNPs in CSSs to that of the flanking sequences. A significant difference in the distribution of DAFs was observed between CSSs and their flanking sequences with, as shown in Fig. 2, a strong shift of DAFs towards lower frequencies was observed in CSSs compared to their flanking sequences. An excess of rare derived alleles (i.e., DAF ≤ 10%) of SNPs was observed in all four HapMap populations: Yoruba in Ibadan, Nigeria (YRI), Japanese in Tokyo, Japan (JPT), Han Chinese in Beijing, China (CHB), and Utah Residents with Northern and Western European Ancestry (CEU). In the human populations YRI, CHB, and CEU, the enrichment of rare derived alleles of SNPs with DAF ≤ 10% is significantly different between CSSs and their flanking sequences (P < 0.05, CHI-Square test). For JPT, the fraction difference of SNPs with DAF ≤ 10% is not statistically significant between CSSs and their flanking sequences (P = 0.06, CHI-Square test), but it is still statistically significant when considering SNPs with DAF ≤ 20% (P < 0.05, CHI-Square test). The CSSs of the YRI population had the highest fraction (0.35) of rare derived alleles with a DAF ≤ 10%, compared to a fraction of 0.29 in the flanking sequences. The CEU population had the greatest fraction difference (0.11) between the fractions of rare derived alleles with DAF ≤ 10% in the CSSs (0.33) compared to their flanking sequences (0.22). This result is similar to that previously reported by Drake *et al*., who documented a strong downward shift of DAFs in conserved compared to nonconserved sequences [23], however, we observed a higher fractional difference (0.06 for YRI) of SNPs with a DAF ≤ 10% between CSSs and their flanking sequences than that (0.03 for YRI) previously documented between conserved elements and nonconserved sequences [23]. The difference between these analyses may be partially attributed to differences in the SNP data set that were used, or given the greater difference in the fraction of SNPs with DAF ≤ 10% observed in our studies, could reflect a stronger intensity of selection against CSSs compared to typical conserved elements. Our observations indicate that CSSs are the most conserved elements, and that they are under extreme strong evolutionary constraints. We then examined the DAFs of sites within the conserved elements that we had previously classified as sites being in stems or loops/bugles. As with SNP density, a downward shift in the DAFs of SNPs was observed in stems compared to loops/bulges (Fig. 3), with the difference showing even greater significance than that observed between CSSs and their flanking sequences. The minimal difference of the fraction of SNPs with DAF ≤ 10% was > 0.09 between the stems and the loops/bulges, except for the JPT population. Variance may exist in this comparison owing to the small number of SNPs in stems and loops/bulges, however, SNPs with DAF ≤ 20% are still significantly enriched in stems than in loops/bulges (P < 0.05, CHI-Square test). Our data from both SNP density and the distribution of DAFs suggests that sites in the stems of CSSs are under stronger evolutionary constraint than sites in loops/bulges, which are still under stronger constraints than that observed in the flanking non-conserved sequences.

### Predicting intergenic CSS-gene associations using genetic distance

We then investigated the associations between intergenic CSSs and their flanking genes. Genetic distance is roughly proportional to physical distance and it appears to be reasonable to hypothesize that non-homologous recombination is less likely to happen between an intergenic CSS and its target gene, thus genetic distance, rather than physical distance, was used to measure the tightness of association between intergenic CSSs and their flanking genes. The genetic distances between intergenic CSSs and their flanking genes was calculated using data for the recombination rates between SNPs spanning the interval where the genes were located. The gene that showed the minimum genetic distance from the intergenic CSS was concluded to be the target gene of the CSS. Given this assumption, intergenic CSSs were found to be enriched near genes encoding transcription factors (P = 1.4 × 10^-5^, CHI-Square test), an observation consistent with previous reports [3,11]. In total, 1069 of the 16,574 protein coding genes annotated in the Gene Ontology (GO) and Gene Ontology Annotation (GOA) databases are associated with intergenic CSSs, and of these 1069 genes, 323 encode transcription factors, constituting a fraction (0.30) much higher than the fraction (of 0.15, 2525/16574) in the annotated GO/GOA databases (P < 0.001, CHI-Square test). Enrichment of CSSs around transcription factor-encoding genes suggests that a substantial portion of intergenic CSSs may function as cis-regulatory elements. In addition, intergenic CSSs were also found to be statistically enriched proximal to genes that are involved in development and differentiation (P < 0.01, CHI-Square test). Detailed results are given in Additional file 1. Strikingly, 138 of the 323 transcription factor-encoding genes associated with CSSs are also known to be important in the development, a fraction (0.43) that is significantly higher than the fraction (0.18, 404/2202) of the remaining transcription factor-encoding genes that are involved in the development but that are not associated with intergenic CSSs (P = 2 × 10^-23^, CHI-Square test). These observations suggest that transcription factor-encoding genes associated with intergenic CSSs regulate developmental processes.

**Figure 4:**
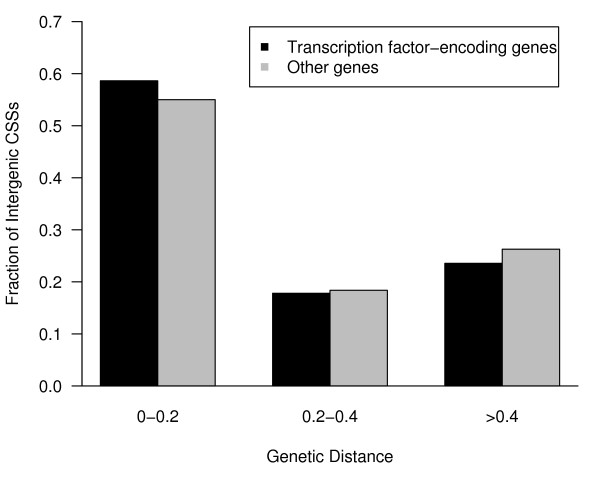
**Distribution of genetic distances between CSSs and associated genes**. Genes associated with CSSs were classified as either transcription factor-encoding or other protein coding genes. About 59% of the CSSs associated with transcription factor-encoding genes are located within a distance ≤0.2 cM, compared to 55% of the CSSs associated with other genes.

**Figure 5:**
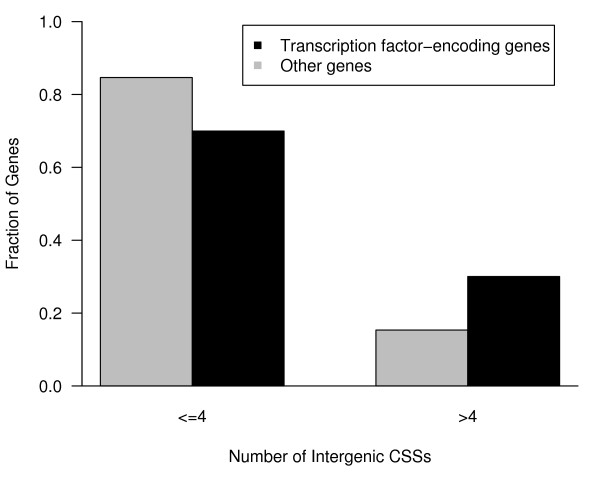
**Comparison of distribution of the number of intergenic CSSs associated with transcription factor-encoding and other genes**. The fraction of transcription factor-encoding and other protein coding genes that have four or less (<= 4) or more than 4 (>4) CSSs are shown. About 30% of the transcription factor-encoding genes, compared to about 15% of other genes, are associated with > 4 intergenic CSSs.

To further investigate the associations between intergenic CSSs and transcription factors-encoding genes, we classified CSS associated genes into two groups: (1) genes encoding transcription factors and (2) genes encoding other proteins. We compared the genetic distance between intergenic CSSs and the genes for these two groups. The average genetic distance between intergenic CSSs and transcription factor-encoding gene is about 0.28 cM (centi-Morgan), which is lower than the average distance (0.33 cM) for the other genes. Fig. 4 shows the distribution of genetic distances between CSSs and the two groups of genes. A larger fraction of the CSSs associated with transcription factor genes than for the other genes (958/1634 or 59% compared to 1505/2736 or 55%, respectively; P < 0.05, CHI-Square test) are located in close proximity of ≤0.2 cM to the genes. We next examined the number of intergenic CSSs that are associated with each gene. On average, each of the 1069 genes that are associated with intergenic CSSs is associated with 3.58 intergenic CSSs. Strikingly a higher fraction of transcription factor-encoding genes were observed to be associated with a larger number of intergenic CSSs. For the 50 genes with highest number of intergenic CSSs, 28 (56%) of them encode transcription factors, a much higher percentage than the 30% (323/1069) that they make up of the number of intergenic CSS associated genes. While the majority of genes are associated with 4 or fewer intergenic CSSs, as shown in Fig. 5, a statistically significant higher fraction of transcription factor-encoding genes are associated with greater than four intergenic CSSs compared to other genes (P < 10^-7^, CHI-Square test). These observations suggest a linkage between intergenic CSSs and transcription factor encoding genes.

### Overlap with transcription factor binding sites

The clustering of a substantial portion of the intergenic CSSs to the proximity of transcription factors-encoding genes is similar to the organization of transcriptional regulation networks that regulate many transcription factor genes [25-27]. For example, Boyer *et al*. have experimentally identified the binding sites for several important transcription factors that affect stem cell identity, including OCT4, SOX2, and NANOG [25], and the binding sites of these three transcription factors are found proximal to many transcription factor genes, including themselves, and therefore may form self-regulatory network loops. In the TFCONES database, a considerable fraction of conserved elements were annotated overlapping with the binding sites of many transcription factors [28]. Here we examined how many of the intergenic CSSs are potentially bound by these important transcription factors. When chromosomal coordinates were used to map transcription factor binding sites and intergenic CSSs, 15, 17, and 18 intergenic CSSs were found to overlap with binding sites for SOX2, OCT4, and NANOG, respectively. The 15 intergenic CSSs potentially bound by SOX2 associate with 14 protein coding genes, of which 8 encode transcription factors. Similarly, 13 and 16 protein coding genes associate with intergenic CSSs bound by OCT4 and NANOG, respectively, of which 10 and 12, respectively, are transcription factor-encoding genes. We also examined whether there was an overlap between intergenic CSSs and the binding sites for C-MYC and SUZ12, factors for which binding sites have also been experimentally mapped [26,27]. We found that 174 (3.86% of the total) intergenic CSSs overlap with binding sites for SUZ12 and 9 (0.20% of the total) overlap binding sites for C-MYC. The 174 intergenic CSSs bound by SUZ12 are associated with 100 genes, 67 of which are encoding transcription factors, while the 9 intergenic CSSs bound by C-MYC are associated with 7 genes, 5 of which are transcription factor-encoding genes. The overlap with binding sites for these five transcription factors indicates that a substantial number of intergenic CSSs may function at the experimentally verified binding sites for these transcription factors.

**Figure 6:**
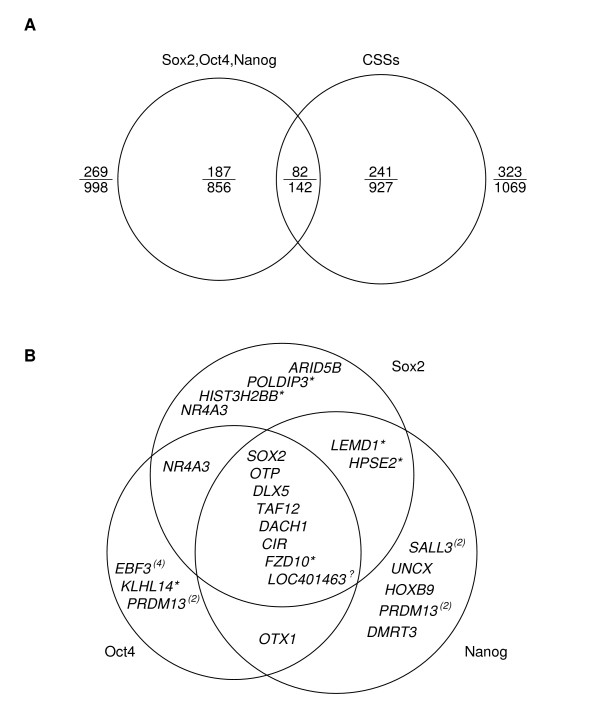
**Venn diagrams showing genes associated with intergenic CSSs and transcription factors SOX2, OCT4, and NANOG**. (A) Overlap between CSS-associated genes and genes regulated by transcription factors SOX2, OCT4, and NANOG. The denominator of each fraction indicates the number of total genes, and the numerator indicates the number of genes that encode transcription factors. A total of 142 genes are both associated with intergenic CSSs and proximal to binding sites for the three transcription factors, and 82 of them encode transcription factors. (B) Genes associated with CSSs are classified as to whether they are adjacent to binding sites for SOX2, OCT4, and NANOG. Genes denoted with an asterisk are not transcription factor-encoding genes and the gene denoted with a question mark has unknown function. For most of the genes, only one of the associated intergenic CSSs could be bound by corresponding transcription factors, except for genes denoted with parenthesis within which the number indicates the number of associated CSSs that could be bound by corresponding transcription factors.

As presented in Fig. 6A, of genes proximal to sequences bound by SOX2, OCT4, and NANOG [25], 998 have been annotated in the GO/GOA databases, with 269 of them encoding transcription factors. For genes that are both associated with intergenic CSSs and proximal to sequences bound by SOX2, OCT4, and NANOG, regardless of whether the intergenic CSSs bind these three transcription factors, 142 have been annotated, of which 82 are encoding transcription factors. If transcription factor binding sites are independent of the existence of CSSs, then the expected probability for each of the 142 genes to be a transcription factor-encoding gene is equal to 0.49 (1-(1-269/998)*(1-323/1069)). The probability that we would have observed at least 82 transcription factor-encoding genes out of the 142 genes is 0.023 (P(82 ≤ x ≤ 142) for distribution Binomial(142, 0.49)), indicating that transcription factor-encoding genes are significantly enriched in the set of genes that are both associated with intergenic CSSs and proximal to sequences bound by the three transcription factors. Only a small number of the genes have CSSs that also act as binding sites for SOX2, OCT4, and NANOG and these are shown in Fig. 6B. Of the 2260 target genes of SOX2, OCT4, and NANOG, only 353 are co-regulated by all three transcription factors [25]. In contrast, a significantly higher fraction, over one-third (8 of 22; P < 0.05, CHI-Square test) of the genes shown in Fig. 6B that have CSSs that bind SOX2, OCT4, and NANOG are co-regulated by all three transcription factors. These 8 genes, *SOX2*, *OTP*, *DLX5*, *DACH1*, *CIR*, *TAF12*, *FZD10*, and *LOC401463*, not only are associated with CSSs that overlap the binding sites for SOX2, OCT4, and NANOG, but also only a single CSS proximal to each gene is bound by all three transcription factors. Thus, for all 8 genes the three transcription factors potentially interact on a single CSS to regulate the expression of a gene. Of these 8 genes, 6 encode transcription factors, while *FZD10 *encodes a protein functioning in the Wnt receptor signaling pathway (that plays roles in pluripotency and self-renewal in embryonic stem cells [29]), and the gene *LOC401463 *encodes a protein of unknown function.

## Discussion

In this study, we have conducted a systematic survey of the evolutionary constraints acting upon CSSs and investigated a relationship between the enrichment of intergenic CSSs and the enrichment in the binding sites for transcription factors proximal to genes that encode transcription factors. Our survey of the evolutionary constraints unveiled that intensive purifying selection acts against CSSs and has favored the maintenance of secondary structures, implying that there is a functional importance to the secondary structures in these conserved sequences. The enrichment of intergenic CSSs near transcription factor-encoding genes suggests that these CSSs likely function as cis-regulatory elements rather than being transcribed into RNAs, since it is not necessary for RNA genes to be organized predominantly near any class of protein coding genes in the genome.

A recent study focusing on a secondary DNA structure near the gene *Hoxb9 *revealed that a DNA secondary structure functions as an important binding site for the protein FBXL10 and this structure is conserved between human and mouse [30]. For the *Hoxb9 *promoter, DNA fragments with two conformations were isolated with identical DNA sequence, one linear and the other containing a secondary structure. Intriguingly, protein FBXL10 exhibits a high binding affinity for the structured promoter, rather than for the linear promoter sequence, strongly suggesting that this protein's binding activity is structure-dependent [30]. Similarly, in this study we have observed an overlap between intergenic CSSs and the binding sites for several transcription factors, which may be due to a steric requirement during the interaction between these transcription factors and genomic DNA sequences. However, protein binding to intergenic CSSs may still be sequence-dependent, since complementary substitutions in an inverted repeat, which retained secondary structure, of the *Hlx *gene promoter did not restore promoter activity[31], thus explaining why CSSs are highly conserved both in structures and in primary sequences. In the case of *Hoxb9*, the protein FBXL10 binds competitively to the structured promoter and the binding is critical to reduce the expression of *Hoxb9 *[30]. In our analysis we found several intergenic CSSs which could each bind at least 2 different transcription factors, suggesting that competitive binding of transcription factors to a single intergenic CSS could occur at these sites. We found 8 intergenic CSSs that are associated with genes that are co-regulated by three transcription factor SOX2, OCT4, and NANOG (see Fig. 6B), where 6 of the CSSs are completely composed of binding site for each of these three transcription factors. Of these six intergenic CSSs, one is also composed of a binding site for SUZ12 (data not shown). Interestingly, Lee *et al*. has previously documented that the expression of genes regulated by SUZ12 changed from expressed to repressed, possibly due to the competitive binding of SUZ12 to cis-regulatory sequences that were previously bound by other transcription factors that activated gene expressions [26]. Many intergenic CSSs may act in a similar manner and function as a switch due to the alternative binding of transcription factors directly affecting the temporal expression of target genes.

**Figure 7:**
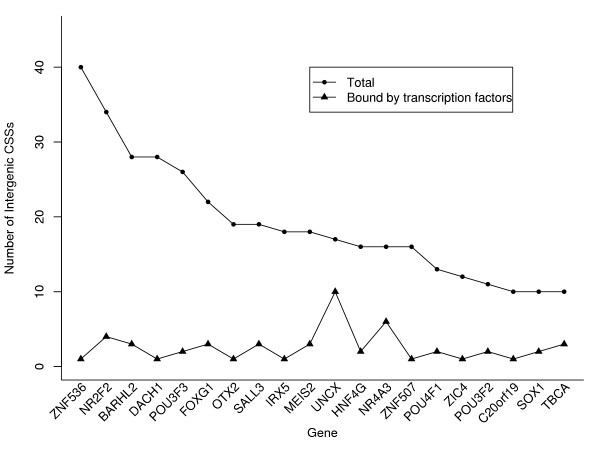
**SOX2, OCT4, NANOG, C-MYC, and SUZ12 only bind to a small fraction of intergenic CSSs**. The 20 genes with highest number of intergenic CSSs, at least one of which overlaps a binding site for the transcription factors SOX2, OCT4, NANOG, C-MYC, and SUZ12. Except for *UNCX*, more than half of the intergenic CSSs for each of these genes are not bound by the studied transcription factors.

As global genomic data on DNA binding is available for only a few transcription factors, it seems likely that a greater fraction of intergenic CSSs will be found to overlap with binding sites for transcription factors. In Fig. 7, we list 20 genes which have the largest numbers of intergenic CSSs, with at least one of the intergenic CSSs binding OCT4, SOX2, NANOG, SUZ12, or C-MYC. The majority of intergenic CSSs for these genes are not bound by any of the five intensively studied transcription factors, except for the gene *UNCX*. It seems plausible that a gene that is associated with many intergenic CSSs could be functionally important, because its normal function may have a restricted expression pattern (either spatial or temporal), which is due to the complex binding of a combination of transcription factors to its neighboring intergenic CSSs. In particular, transcription factors-encoding genes that regulate developmental processes were found to be significantly enriched as target genes of intergenic CSSs, and these are genes where ectopic expression leads to unexpected and harmful results. For example, misexpression of *Ultrabithorax *and *abdominal-A*, two HOX genes determining segmental identities, transform *Drosophila *antennal tissue into leg tissue and wing tissue into halter tissue respectively [32]. The characterization of neighboring intergenic CSSs should advance our insight into the biological functions of the target genes.

Our study supports the hypothesis that DNA secondary structures are important units that function in the interaction between proteins and genomic DNA sequences. Investigating mutations that change the paring status of CSSs should facilitate the identification of functional variants that predispose to genetic diseases.

## Conclusion

Despite the evolutionary conservation of conserved secondary structures (CSSs) a considerable amount of variation in CSS sequences exist in the genomes of human populations. Analyses of the variant sequences that exist in human populations demonstrate that sites in stems of the predicted secondary structures are under stronger evolutionary constraint than sites on loops/bulges, which are still more constrained than non-conserved sequences. An overlap between CSSs and the binding sites of transcription factors was found to be enriched near transcription factor-encoding genes suggesting a role for CSSs in transcriptional regulation networks.

## Methods

Human genomes (assembly hg18 and hg17) were downloaded from the UCSC Genome Bioinformatics Site [33]. CSSs were retrieved from the EvoFold home page [20], but only long CSSs (at least 15 pairing bases) were used in this analysis. An application using hash-tables was written to map CSSs from human genome hg17 to hg18. Locations and SNP ancestral allele data were obtained from the dbSNP database available at the NCBI [34]. Frequencies of SNPs in the HapMap populations: Yoruba in Ibadan, Nigeria (YRI), Japanese in Tokyo, Japan (JPT), Han Chinese in Beijing, China (CHB), and Utah Residents with Northern and Western European Ancestry (CEU), and the recombination rates between SNPs were derived from the HapMap project (Release #22) [35]. Information of genes was retrieved from the GenBank database [34]. Genes were categorized into different functional groups according to the annotations in the Gene Ontology database (GO) [36] and in the Gene Ontology Annotation database (GOA) [37]. Binding sites for transcription factors SOX2, OCT4, NANOG, C-MYC, and SUZ12 were downloaded as described from previous reports [25-27]. Positions of SNPs relative to CSSs and the overlap between CSSs and the binding sites of transcription factors were calculated according to their chromosomal coordinates in the human genome. SNP densities of flanking sequences were calculated 50 times on each side of the CSSs with a moving window of 200 bp in size. DAFs for SNPs were calculated using ancestral alleles. Genetic distances between the intergenic CSSs and flanking genes was obtained by calculating the genetic distance between two SNPs spanning the genomic interval having the minimal physical distance. The target gene of a CSS was chosen as the flanking gene that showed the minimum genetic distance to the intergenic CSS. Statistical analyses were performed and figures were prepared by using the R software [38].

## Abbreviations

RS: rejected substitutions; CSS: conserved secondary structure; DAF: derived allele frequency; GO: Gene Ontolgy; GOA: Gene Ontology Annotation; YRI: Yoruba in Ibadan, Nigeria; JPT: Japanese in Tokyo, Japan; CHB: Han Chinese in Beijing, China; CEU: Utah Residents with Northern and Western European Ancestry.

## Authors' contributions

HBX and YPZ conceived and designed the project. HBX performed the comparison work, analyzed the results, and wrote the paper. Both YPZ and DMI gave many suggestions for the detailed analysis, contributed to the discussions, and improved the paper.

## Supplementary Material

Additional file 1 **Gene categories strongly associated with intergenic CSSs.** Click here for file

## References

[B1] Cooper GM, Stone EA, Asimenos G, Green ED, Batzoglou S, Sidow A (2005). Distribution and intensity of constraint in mammalian genomic sequence. Genome research.

[B2] Dermitzakis ET, Reymond A, Scamuffa N, Ucla C, Kirkness E, Rossier C, Antonarakis SE (2003). Evolutionary discrimination of mammalian conserved non-genic sequences (CNGs). Science.

[B3] Bejerano G, Pheasant M, Makunin I, Stephen S, Kent WJ, Mattick JS, Haussler D (2004). Ultraconserved elements in the human genome. Science.

[B4] Ovcharenko I, Stubbs L, Loots GG (2004). Interpreting mammalian evolution using Fugu genome comparisons. Genomics.

[B5] Margulies EH, Blanchette M, Haussler D, Green ED (2003). Identification and characterization of multi-species conserved sequences. Genome research.

[B6] Siepel A, Bejerano G, Pedersen JS, Hinrichs AS, Hou M, Rosenbloom K, Clawson H, Spieth J, Hillier LW, Richards S, Weinstock GM, Wilson RK, Gibbs RA, Kent WJ, Miller W, Haussler D (2005). Evolutionarily conserved elements in vertebrate, insect, worm, and yeast genomes. Genome research.

[B7] Bird CP, Stranger BE, Dermitzakis ET (2006). Functional variation and evolution of non-coding DNA. Curr Opin Genet Dev.

[B8] Dermitzakis ET, Reymond A, Antonarakis SE (2005). Conserved non-genic sequences – an unexpected feature of mammalian genomes. Nat Rev Genet.

[B9] Kamal M, Xie X, Lander ES (2006). A large family of ancient repeat elements in the human genome is under strong selection. Proceedings of the National Academy of Sciences of the United States of America.

[B10] Tran T, Havlak P, Miller J (2006). MicroRNA enrichment among short 'ultraconserved' sequences in insects. Nucleic Acids Res.

[B11] Woolfe A, Goodson M, Goode DK, Snell P, McEwen GK, Vavouri T, Smith SF, North P, Callaway H, Kelly K, Walter K, Abnizova I, Gilks W, Edwards YJ, Cooke JE, Elgar G (2005). Highly conserved non-coding sequences are associated with vertebrate development. PLoS Biol.

[B12] Plessy C, Dickmeis T, Chalmel F, Strahle U (2005). Enhancer sequence conservation between vertebrates is favoured in developmental regulator genes. Trends Genet.

[B13] Frazer KA, Tao H, Osoegawa K, de Jong PJ, Chen X, Doherty MF, Cox DR (2004). Noncoding sequences conserved in a limited number of mammals in the SIM2 interval are frequently functional. Genome research.

[B14] Loots GG, Locksley RM, Blankespoor CM, Wang ZE, Miller W, Rubin EM, Frazer KA (2000). Identification of a coordinate regulator of interleukins 4, 13, and 5 by cross-species sequence comparisons. Science.

[B15] Vavouri T, McEwen GK, Woolfe A, Gilks WR, Elgar G (2006). Defining a genomic radius for long-range enhancer action: duplicated conserved non-coding elements hold the key. Trends Genet.

[B16] Sun H, Skogerbo G, Chen R (2006). Conserved distances between vertebrate highly conserved elements. Hum Mol Genet.

[B17] Lettice LA, Heaney SJ, Purdie LA, Li L, de Beer P, Oostra BA, Goode D, Elgar G, Hill RE, de Graaff E (2003). A long-range Shh enhancer regulates expression in the developing limb and fin and is associated with preaxial polydactyly. Hum Mol Genet.

[B18] Beysen D, Raes J, Leroy BP, Lucassen A, Yates JR, Clayton-Smith J, Ilyina H, Brooks SS, Christin-Maitre S, Fellous M, Fryns JP, Kim JR, Lapunzina P, Lemyre E, Meire F, Messiaen LM, Oley C, Splitt M, Thomson J, Peer Y Van de, Veitia RA, De Paepe A, De Baere E (2005). Deletions involving long-range conserved nongenic sequences upstream and downstream of FOXL2 as a novel disease-causing mechanism in blepharophimosis syndrome. Am J Hum Genet.

[B19] Loots GG, Kneissel M, Keller H, Baptist M, Chang J, Collette NM, Ovcharenko D, Plajzer-Frick I, Rubin EM (2005). Genomic deletion of a long-range bone enhancer misregulates sclerostin in Van Buchem disease. Genome research.

[B20] Pedersen JS, Bejerano G, Siepel A, Rosenbloom K, Lindblad-Toh K, Lander ES, Kent J, Miller W, Haussler D (2006). Identification and classification of conserved RNA secondary structures in the human genome. PLoS Comput Biol.

[B21] Glazov EA, Pheasant M, McGraw EA, Bejerano G, Mattick JS (2005). Ultraconserved elements in insect genomes: a highly conserved intronic sequence implicated in the control of homothorax mRNA splicing. Genome research.

[B22] Blanchette M, Kent WJ, Riemer C, Elnitski L, Smit AF, Roskin KM, Baertsch R, Rosenbloom K, Clawson H, Green ED, Haussler D, Miller W (2004). Aligning multiple genomic sequences with the threaded blockset aligner. Genome research.

[B23] Drake JA, Bird C, Nemesh J, Thomas DJ, Newton-Cheh C, Reymond A, Excoffier L, Attar H, Antonarakis SE, Dermitzakis ET, Hirschhorn JN (2006). Conserved noncoding sequences are selectively constrained and not mutation cold spots. Nat Genet.

[B24] Fay JC, Wyckoff GJ, Wu CI (2001). Positive and negative selection on the human genome. Genetics.

[B25] Boyer LA, Lee TI, Cole MF, Johnstone SE, Levine SS, Zucker JP, Guenther MG, Kumar RM, Murray HL, Jenner RG, Gifford DK, Melton DA, Jaenisch R, Young RA (2005). Core transcriptional regulatory circuitry in human embryonic stem cells. Cell.

[B26] Lee TI, Jenner RG, Boyer LA, Guenther MG, Levine SS, Kumar RM, Chevalier B, Johnstone SE, Cole MF, Isono K, Koseki H, Fuchikami T, Abe K, Murray HL, Zucker JP, Yuan B, Bell GW, Herbolsheimer E, Hannett NM, Sun K, Odom DT, Otte AP, Volkert TL, Bartel DP, Melton DA, Gifford DK, Jaenisch R, Young RA (2006). Control of developmental regulators by Polycomb in human embryonic stem cells. Cell.

[B27] Zeller KI, Zhao X, Lee CW, Chiu KP, Yao F, Yustein JT, Ooi HS, Orlov YL, Shahab A, Yong HC, Fu Y, Weng Z, Kuznetsov VA, Sung WK, Ruan Y, Dang CV, Wei CL (2006). Global mapping of c-Myc binding sites and target gene networks in human B cells. Proceedings of the National Academy of Sciences of the United States of America.

[B28] Lee AP, Yang Y, Brenner S, Venkatesh B (2007). TFCONES: a database of vertebrate transcription factor-encoding genes and their associated conserved noncoding elements. BMC Genomics.

[B29] Sato N, Meijer L, Skaltsounis L, Greengard P, Brivanlou AH (2004). Maintenance of pluripotency in human and mouse embryonic stem cells through activation of Wnt signaling by a pharmacological GSK-3-specific inhibitor. Nat Med.

[B30] Yamagishi T, Hirose S, Kondo T (2008). Secondary DNA structure formation for Hoxb9 promoter and identification of its specific binding protein. Nucleic Acids Res.

[B31] Bates MD, Schatzman LC, Harvey RP, Potter SS (2001). Two CCAAT boxes in a novel inverted repeat motif are required for Hlx homeobox gene expression. Biochimica et biophysica acta.

[B32] Casares F, Calleja M, Sanchez-Herrero E (1996). Functional similarity in appendage specification by the Ultrabithorax and abdominal-A Drosophila HOX genes. EMBO J.

[B33] The UCSC Genome Bioinformatics Site. http://genome.ucsc.edu.

[B34] NCBI. http://www.ncbi.nlm.nih.gov.

[B35] The HapMap Project. http://www.hapmap.org.

[B36] The Gene Ontology Database. http://www.geneontology.org.

[B37] The Gene Ontology Annotation Database. http://www.ebi.ac.uk/GOA.

[B38] R Development Core Team (2008). R: A language and environment for statistical computing.

